# Predictors of mortality and survival probability distribution among patients on tuberculosis treatment in Vihiga County, Kenya

**DOI:** 10.4314/ahs.v23i1.24

**Published:** 2023-03

**Authors:** Paul Waliaula Wekunda, Dickens S Omondi Aduda, Bernard Guyah, James Odongo

**Affiliations:** 1 Department of Health; Vihiga County Government, Kenya; 2 School of Health Sciences: Directorate of Research, Innovation and Partnerships; Jaramogi Oginga Odinga University of Science and Technology; 3 Department of Biomedical Sciences; Maseno University; 4 Department of Mathematics and applied sciences; Ramogi Institute of Advanced Technology

**Keywords:** TB mortality, survival distributions, treatment outcomes, Vihiga

## Abstract

**Background:**

Tuberculosis (TB) related mortality remains a serious impediment in ending TB epidemic.

**Objective:**

To estimate survival probability and identify predictors, causes and conditions contributing to mortality among TB patients in Vihiga County.

**Methods:**

A cohort of 291 patients from 20 purposively selected health facilities were prospectively considered. Data was obtained by validated questionnaires through face-to-face interviews. Survival probabilities were estimated using Kaplan-Meier method while Cox proportional hazard model identified predictors of TB mortality through calculation of hazard ratios at 95% confidence intervals. Mortality audit data was qualitatively categorized to elicit causes and conditions contributing to mortality.

**Results:**

209 (72%) were male, median age was 40 (IQR=32-53) years while TB/HIV coinfection rate was 35%. Overall, 45 (15%) patients died, majority (78% (log rank<0.001)) during intensive phase. The overall mortality rate was 32.2 (95% CI 23.5 - 43.1) deaths per 1000 person months and six months' survival probability was 0.838 (95% CI, 0.796-0.883). Mortality was higher (27%) among HIV positive than HIV negative (9%) TB patients. Independent predictors of mortality included; comorbidities (HR = 2.72, 95% CI,1.36–5.44, p< 0.005), severe illness (HR=5.06, 95% CI,1.59–16.1, p=0.006), HIV infection (HR=2.56, 95% CI,1.28–5.12, p=0.008) and smoking (HR=2.79, 95% CI,1.01–7.75, p=0.049). Independent predictors of mortality among HIV negative patients included; comorbidities (HR = 4.25, 95% CI; 1.15-15.7, p = 0.03) and being clinically diagnosed (HR = 4.8, 95% CI; 1.43-16, P = 0.01) while among HIV positive; they included smoking (HR = 4.05, 95% CI;1.03-16.0, P = 0.04), severe illness (HR = 5.84, 95% CI; 1.08-31.6, P = 0.04), severe malnutrition (HR = 4.56, 95% CI; 1.33-15.6, P = 0.01) and comorbidities (HR = 3.04, 95% CI; 1.03-8.97, p = 0.04). More than a half (52%) of mortality among HIV positive were ascribed to advanced HIV diseases while majority of (72%) of HIV negative patients died to TB related lung disease. Conditions contributing to mortality were largely patient and health system related.

**Conclusion:**

Risk of TB mortality is high and is attributable to comorbidities, severe illness, HIV and smoking. Causes and conditions contributing to TB mortality are multifaceted but modifiable. Improving TB/HIV care could reduce mortality in this setting.

## Introduction

Whereas tuberculosis (TB) is preventable and curable, it remains a leading cause of mortality from single infectious disease agent 1. In 2019, an estimated 10 million people fell ill with TB globally and a total of 1.7 million died 2. More than two thirds of TB cases occur in southeast Asia (44%) and Africa (25%) while lower incidences are observed in the Eastern Mediterranean region (7%), the European region (3%) and the region of the Americas (3%) 2. Although Kenya has been removed from the list of high burden drug resistant TB countries, it remains one of 30 high TB and TB/HIV burden countries globally, with Gabon and Uganda joining the list 3. Kenya, has an overall TB prevalence of 426 per 100,000 population 4 and the disease is responsible for 6.3% annual deaths and total Disability-Adjusted Life years (DALYs) of 4.8% 1,5. Although Kenya reported a slight increase in treatment success rate (TSR) for all forms of TB from 82.4% for the 2017 cohort to 84% for 2018 cohort 6, these rates are still below global TSR target of 90%. Additionally, there was an increase in TB death rate from 6.3% to 6.5% in 2017 and 2018 cohorts respectively. During the same period, 13% of TB patients died in Vihiga County, making it second among counties with highest TB related mortality in Kenya^7^. Understanding factors associated with increased risks of mortality is important in prioritization of key interventions and target groups.

The WHO's ‘End TB Strategy’ and the United Nation's Sustainable Development Goals (SDGs) share a common goal of ending global TB epidemic 8,9. The milestones and targets include a 90% reduction in TB deaths and 80% reduction in TB incidence by 2030 and 95% reduction in TB deaths and 90% reduction in TB incidence by 2035 compared with 2015 figures 9,10. However, the sluggish progress towards achieving these targets across high TB burden countries such as Kenya remains the biggest hurdle^11^. This is despite rigorous intervention programs such as use of molecular and culture methods for TB diagnosis, use of short-course fixed dose combination drugs, nutritional support, TB/HIV collaboration and routine follow up of patients 12. Although several studies 13–17 concur on the need for a person-centered intervention integrated within a multi-sectoral strategy, there still exists scanty information on reasons for often observed survival distributions and causes of increased risk for mortality among patients on TB treatment. Such information is useful for more accurate prediction of risks and occurrence of mortality.

Previous studies have associated TB mortality with female gender 13, male gender 14, bacteriological unconfirmed TB, advanced age 15, comorbidities 16,18,19, behavioral characteristics 16,20 and HIV 17,21,22. However, routine surveillance data may not provide sufficient variables to assess survival probably patterns and analyse the influence of broader demographic, socio-economic and clinical factors on mortality of TB patients 23,24 hence may not be applicable to the local setting. Through prospective follow up design, this study evaluated survival probability distributions and identified predictors, causes and conditions contributing to mortality among TB patients in Vihiga County. Sub group analysis for HIV positive and HIV negative patients was also conducted. Findings of this study have implication of better understanding TB epidemiology and accurate prediction of risks and occurrence of mortality. This can permit cost effective intervention strategies and heightened surveillance in high TB burden settings.

## Methods and procedures Setting

The current study was conducted in Vihiga county which is located in Western region of Kenya and has a population of 590,013 25. The county has consistently reported high rates of mortality among patients on TB treatment^6^,^7^,^26^, hence it provided a robust context for the present study. The county has four TB treatment zones; Emuhaya, Vihiga, Sabatia and Hamisi. This study was conducted among TB patients diagnosed and followed up in twenty selected health facilities which account for 85% of TB cases annually in the county 6,7,26.

### Diagnosis, treatment and follow up of drug susceptible TB

In Kenya, TB diagnosis, treatment and follow up is guided by the integrated guideline 12. Bacteriological confirmation of TB is achieved through molecular, phenotypic and radiological techniques. All cases of drug susceptible TB except TB affecting meninges or bones and drug resistant TB are treated with chemotherapy comprising of two months (intensive phase) with rifampicin (R), isoniazid (H), pyrazinamide (Z) and ethambutol (E) (RHZE) followed by four months (continuation phase) with rifampicin (R) and isoniazid (H) (RH). After preliminary assessment of patients, drugs are administered orally through fixed dose combination and the dosage is determined by patients' body weight. Most patients are treated on ambulatory basis. Patients are required to visit health facilities weekly during intensive phase and twice monthly during continuation phase, during which they are assessed, counseled and given the medicine. Treatment outcome is assigned immediately after treatment completion or after occurrence of an event that constitutes termination of treatment such as death or treatment interruption.

### Study participants

This study included TB patients 15 years and older, duly diagnosed with TB, notified in treatment registers and the national electronic data base (TIBU) and started on TB treatment. Patients with TB of the bones and joints, TB meningitis and drug resistant TB were excluded from the study.

### Study design

Observational cohort study was conducted among TB patients in Vihiga County, enrolled between June and December 2019. After obtaining baseline data, each patient was followed up through routine visits until s/he completed treatment, died or was lost to follow up.

### Sample size

Sample size for the current study was calculated using Cochrane equation 27. The accessible population for this study was 850, the average number of notified TB cases annually in Vihiga County between 2012 to 2018^7^,^28^. Since sample size for this study (384) exceeded 5% of the population size (850), a correction formula was calculated [n = 384/ 1+ [(384 – 1)/850] to yield 265. Ten percent (10%) of 265, (26) was added to compensate for anticipated drop-outs, hence the sample size (n) was 291.

### Sampling procedure

The study area and twenty health facilities that account for 85% of TB cases annually were purposively selected, five from each of the four TB treatment zones. The sample was allocated to the four TB treatment zones and then twenty health facilities proportional to their annual contribution of TB patients. Within facilities, simple random sampling method was used to select eligible patients into the study.

### Data collection

Structured questionnaires were administered to eligible TB patients through face-to-face interviews by twenty trained research assistants, each attached to the participating health facility. The questionnaires comprised closed-ended questions covering patient demographic, socio-economic and clinical characteristics. Follow up data and TB treatment outcome information were also included. Additional data focusing on HIV and comorbidities were captured from patients' clinical records while mortality audit data from patients who died was obtained using the national TB mortality audit tool.

### Variable definition

The outcome variable for this study was all cause mortality (death), ‘yes’ or ‘no’. The total follow-up time for each patient was 180 days, number of days from treatment initiation until completion of treatment. Patients who completed treatment and those who interrupted treatment were censored. Time until event occurred was defined as time in days from treatment initiation to death. Predictor variables included patients' demographic, socio-economic and clinical characteristics. Alcohol and smoking statuses were self-reported while alcohol Use Disorder Identification Test (AUDIT) scoring system was used to assess alcohol use 29. Severely ill patients were defined as clinically very sick with at least respiratory rate > 30/min, temperature > 39°C, heart rate > 120/min and unable to walk unaided. Comorbidities in the current study included all other underlying conditions apart from HIV infection.

### Statistical analysis

Quantitative data was uploaded to R for statistical analysis. Standard descriptive statistics such as frequencies, proportions, mean, median, range and standard deviation were calculated to demonstrate the demographic, socioeconomic and clinical characteristics of TB patients and characterize their probability distributions. Survival analysis was used due to their usefulness in handling time to event outcomes and censored observations^30^. Kaplan Meier (KM) estimator was calculated to obtain univariable descriptive statistics for survival data, including estimation of survival probabilities by patient characteristics. The Log rank (Mantel-Cox) test for the equality of survival distributions was used to analyse the significance of survival differences of TB patients by their characteristics. Variables with p value < 0.05 and universal variables such as age and sex were included in multivariable analysis; Cox proportional hazard (CPH) model, was fitted to identify predictors of all-cause mortality through calculation of hazard ratios at 95% confidence interval (CI). Due to high TB/HIV coinfection, difference in mortality and difference in clinical characteristics between HIV positive and HIV negative TB patients, the sample size was broken down into two subgroups based on their HIV status. For subgroup analysis, Kaplan Meier (KM) estimator was calculated to estimate survival probabilities by patient characteristics among HIV positive and HIV negative subgroup. CPH model, was fitted within each sub group to identify predictors of all-cause mortality through calculation of hazard ratios at 95% confidence interval (CI). Before fitting the covariates into the CPH model, proportional hazard assumption was checked by plotting Schoenfeld residuals against time to test for independence between time and residuals. Any covariate that violated the assumption was stratified. For variable analysis p-value <0.05 was considered significant. Data from mortality audit was qualitatively categorized to elicit causes and conditions contributing to mortality.

### Ethics statement

The current study was approved by the Maseno University Ethics Review Committee (MUERC) (Ref: MSU/DRPI/MUERC/00707/19) and the National Commission for Science, Technology & Innovation (NACOSTI) (Ref: 192517) and was conducted according to Helsinki's declaration. Written informed consent was obtained from all participants and confidentiality was ensured throughout the study.

## Results

### Description of the cohort

Of the 291 patients, 209 (72%) were male, median age (inter-quartile range (IQR)) was 40 (32-52) years and 101 (35%) were HIV positive. One-thirty-six (47%) patients reported passive or active smoking, 136 (47%) were severely ill and 36(12%) had underlying comorbidities. Two hundred and twelve patients (73%) successfully completed their treatment, 32 (11%) interrupted treatment (lost to follow up) and 45 (15%) died.

### Incidence of mortality and survival probability distribution

**Figure 1:**
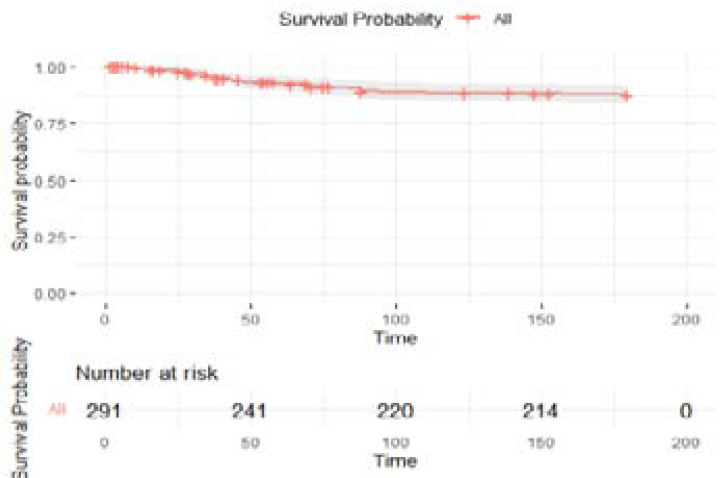
Kaplan Meier curves showing six months' survival probability of TB patients

Of the 291 patients under observation, 45 (15%) patients died comprising a mortality rate of 32.2 (95% CI; 23.5 - 43.1) deaths per 1000 person months. Overall, 78% (log rank = <0.001) of incidences of death occurred during the intensive phase of treatment. The overall survival probability of patients on TB treatment was 0.838 (95% CI, 0.796 - 0.883), Figure 1.

Cumulative incidences of death, 6-months survival probability and survival differences by patients' socio-demographic characteristics are presented in Table 1. Significantly lower survival probability was observed among TB patients who consume alcohol (yes=0.78, no=0.9; Log Rank, p = 0.01) and those who smoke (yes=0.75, no=0.91: Log Rank p = <0.001).

**Table 1:** Cumulative incidences of death, 6-months survival probability and survival differences by patients' socio-demographic characteristics

Socio-demographic characteristics		Sample (n=291)	Died (%)	6-Month survival probability	Log Rank (*p* -value)
Zone	Emuhaya	87	13 (15%)	0.84	0.7 (0.91)
	Hamisi	63	9 (14%)	0.85	
	Sabatia	40	5(13%)	0.85	
	Vihiga	101	18 (18%)	0.81	

Sector	Faith Based	50	11 22%)	0.76	2.4 (0.13)
	Public	241	34 (14%)	0.85	
Age	Median (IQR)	40 (32–53)	45 (34–59)	-	94.5 (0.01)

sex	Female	82	13 (16%)	0.83	0 (0.86)
	Male	209	32 (15%)	0.84	

Pregnancy	NA	209	32 (15%)	0.84	0.2 (0.92)
	No	77	12 (16%)	0.84	
	Yes	5	1 (20%)	0.75	

Occupation	Employed	19	2 (11%)	0.89	0.5 (0.48)
	Not Employed	272	43 (16%)	0.83	

Marital Status	Married	111	14 (13%)	0.87	1.5 (0.22)
	Not married	180	31 (17%)	0.82	

Education	Post-secondary	24	2 (8%)	0.91	2.7 (0.26)
	Primary or Lower	172	31 (18%)	0.81	
	Secondary	95	12 (13%)	0.87	

Duration of illness	1–2 weeks	39	4 (10%)	0.89	5.2 (0.08)
	2–4 weeks	69	6 (9%)	0.91	
	More than four weeks	183	35 (19%)	0.79	

First sector visited	Chemist	73	14 (19%)	0.8	1.8 (0.78)
	Faith based facility	24	4 (17%)	0.83	
	Herbal	10	2 (20%)	0.78	
	Private facility	47	7 (15%)	0.84	
	Public facility	137	18 (13%)	0.86	

Services done	Given drugs	162	24 (15%)	0.84	1.1 (0.57)
	Investigated for TB	126	20 (16%)	0.84	
	Referred	3	1 (33%)	0.67	

Treatment Supporter	No	141	26 (18%)	0.8	2.4 (0.12)
	Yes	150	19 (13%)	0.87	

Smoking	No	155	14 (9%)	0.91	11.3 (<0.001)
	Yes	136	31 (23%)	0.75	

Alcohol consumption	No	135	14 (10%)	0.9	6.5 (0.01)
	Yes	156	31 (20%)	0.78	

Substance abuse	No	260	40 (15%)	0.81	0 (0.91)
	Yes	31	5 (16%)	0.84	

IQR= interquartile range

**Figure 2:**
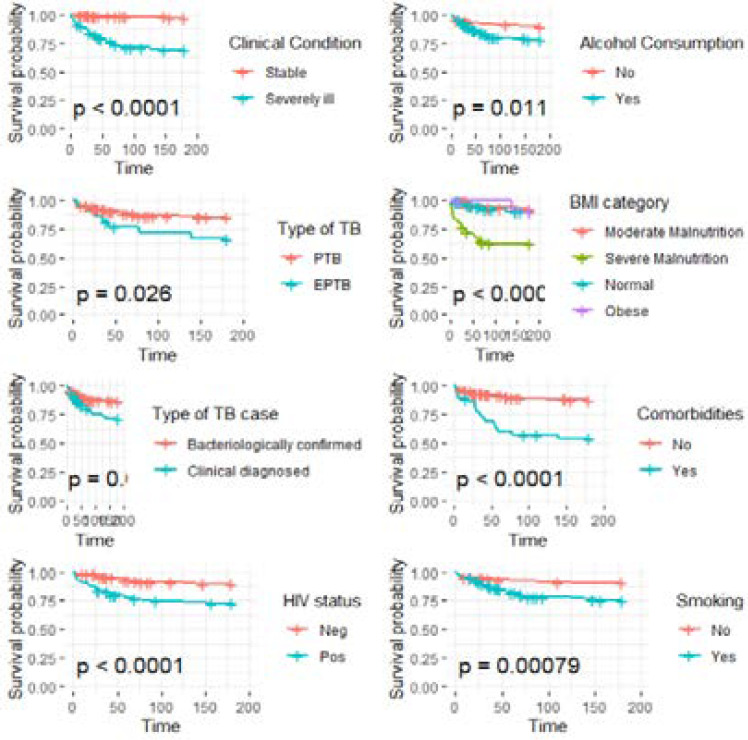
Kaplan Meier curves showing patient factors associated with reduced survival probability

Cumulative incidences of death, 6-months survival probability and survival differences by patients' clinical characteristics are presented in Table 2, while Figure 2 presents Kaplan Meier curves showing patient factors associated with reduced survival probability among TB patients. Significantly lower survival was observed among severely ill patients (severely ill = 0.68, stable = 0.9; Log Rank p <0.001), Extra pulmonary TB (EPTB) (EPTB = 0.67, PTB= 0.85; Log Rank p = 0.03); clinically diagnosed TB patients (clinically diagnosed = 0.72, bacteriologically confirmed = 0.87; Log Rank p = 0.005); HIV infection (pos = 0.72, neg = 0.9; Log Rank p<0.001); severely malnourished (severe malnutrition = 0.62, normal = 0.93, moderate malnutrition = 0.9, overweight = 0.91; Log Rank p <0.001); and having comorbid conditions (no = 0.54, yes = 0.88; Log Rank p <0.001).

**Table 2:** Cumulation incidences of death, 6-months survival probability and survival differences by Patients' clinical characteristics

Clinical Characteristics		Frequency	Died (%)	6-month's survival probability	Log Rank (P-Value)
Clinical Condition	Stable	155	4 (3%)	0.97	42.7(<0.001)
	Severely ill	136	41 (30%)	0.68	

Type of TB	^1^EPTB	22	7 (32%)	0.67	5(0.03)
	PTB	269	38 (14%)	0.85	

Site of EPTB	Lymph Nodes	7	3 (43%)	0.56	6.2(0.10)
	Abdominal	1	0	1	
	Pleural Effusion	14	4 (29%)	0.69	

Classification of TB case	^2^Bact confirmed	227	28 (12%)	0.87	8(0.005)
	Clinically Diagnosed	64	17 (27%)	0.71	

Type of Patient	Failure	2	0	1	0.9(0.81)
	New	246	40 (16%)	0.83	
	Relapse	30	4 (13%)	0.86	
	^3^TLF	13	1 (8%)	0.92	

^4^TPT for HIV positive	ART Naïve	65	13 (20%)	0.81	28.4(<0.001)
	No	14	4 (29%)	0.64	
	Yes	22	10 (45%)	0.54	

HIV Status	Neg	190	18 (9%)	0.9	15.9(<0.001)
	Pos	101	27 (27%)	0.72	

^5^ART uptake	No	4	2 (50%)	0.5	19.7(<0.001)
	Yes	97	25 (26%)	0.73	

Viral Suppression	Not due	67	13 (19%)	0.79	29.7(<0.001)
	Not done	7	2 (29%)	0.71	
	Not Suppressed	18	9 (50%)	0.49	
	Suppressed	9	3 (33%)	0.67	

Ever interrupted ART	No	6	1 (17%)	0.83	27.9(<0.001)
	ART Naïve	65	13 (20%)	0.79	
	Yes	30	13 (43%)	0.56	

^6^BMI category	Moderate Malnutrition	99	11 (11%)	0.9	43(<0.001)
	Normal	105	7 (7%)	0.93	
	Over weight	12	1 (7%)	0.91	
	Severe Malnutrition	75	28 (37%)	0.62	

*Comorbidities	No	255	29 (11%)	0.88	29.7(<0.001)
	Yes	36	16 (44%)	0.54	

1EPTB = Ext rapulmonary TB; 2Bact = Bacteriologically; EPTB Extra pulmonary TB; 3TLF = Treatment after loss to follow up; 4TPT = TB preventive therapy; 5ART = Antiretroviral Therapy; 6BMI = Body Mass Index; *Comorbidities = underlying non-communicable diseases.

### Predictors of all-cause mortality among TB patients

Before fitting the covariates into the multivariable cox model, proportional hazard assumption was checked by plotting Schoenfeld residuals against time to test for independence between time and residual. BMI category was found to significantly (2.1%) differ from zero at the 5% significance level, therefore, the final model was corrected by stratification of “BMI category” covariate. After simultaneously controlling for the potential predictor variables (Table 3), TB patients with underlying comorbidities were almost 3 times more likely to die (HR = 2.72, 95% CI; 1.36–5.44, p = 0.005) compared to patients with- out comorbidities. Besides, patients who were severely ill during treatment initiation were five times more likely to die (HR = 5.06, 95% CI; 1.59–16.1, p = 0.006) compared to clinically stable patients. TB and HIV co-infected patients were two and a half times more likely to die (HR = 2.56, 95% CI; 1.28–5.12, p = 0.008) compared to the HIV negative patients, while those who smoked were almost 3 times more likely to die (HR = 2.79, 95% CI; 1.01–7.75, p = 0.049) compared to non-smokers.

**Table 3:** Predictors of mortality among TB patients

Characteristic		HR^1^	95% CI^1^	p-value
**Age**	Age in years	1.01	0.99, 1.03	0.43

**Sex**	Male	—	—	
	Female	0.7	0.6, 4.82	0.32

**Education level**	Secondary	—	—	
	Primary or lower	0.98	0.46, 2.06	0.95
	Post-secondary	1.19	0.23, 6.26	0.83

**Occupation**	Employed	—	—	
	Not employed	0.47	0.09, 2.41	0.36

**Symptom duration**	1–2 weeks	—	—	
	2–4 weeks	1.23	0.29, 5.33	0.78
	>4 weeks	1.86	0.56, 6.16	0.31

**Alcohol** **consumption**	No	—	—	
	Yes	1.12	0.38, 3.31	0.831

**Smoking**	No	—	—	
	Yes	2.79	1.01, 7.75	0.049*

**Clinical Condition**	Stable	—	—	
	Severely ill	5.06	1.59, 16.1	0.006**

**HIV Status**	Neg	—	—	
	Pos	2.56	1.28, 5.12	0.008**

**Type of TB**	PTB	—	—	
	EPTB	1.33	0.53, 0.31	0.54

**Comorbidities**	No	—	—	
	Yes	2.72	1.36, 5.44	0.005**

1 HR = Hazard Ratio, CI = Confidence Interval, PTB=Pulmonary TB, EPTB=Extra pulmonary TB

### Analysis of HIV positive and HIV negative subgroups

Cumulatively, of 101 TB/HIV coinfected patients 27 (27%) died comprising a mortality rate of 61.7 (95% CI; 40.6 – 89.7) deaths per 1000 person months. Among the 190 HIV negative TB patients, 18 (9%) died comprising mortality rate of 18.7 (95% CI; 11.1 – 29.6) deaths per 1000 person months.

### Univariable and multivariable analysis of HIV negative and HIV positive subgroups

Among HIV negative TB patients, significantly lower survival probability was observed among those who consume alcohol (yes=0.84, no=0.95: Log Rank p = <0.024), smokers (yes=0.82, no=0.96: Log Rank p = <0.005), the severely malnourished (severe malnutrition = 0.68, overweight = 0.88, moderate malnutrition = 0.96; Log Rank p <0.001); severely ill (severely ill = 0.76, clinically stable = 0.96; Log Rank p <0.001), clinically diagnosed (clinically diagnosed = 0.73, bacteriologically confirmed = 0.93; Log Rank p = 0.001), extra pulmonary TB (EPTB) patients (EPTB = 0.67, PTB = 0.91; Log Rank p = 0.01), and those with comorbidities (no = 0.93, yes = 0.58; Log Rank p <0.001). On multivariable analysis, stratified by smoking, HIV negative TB patients with underlying comorbidities were almost 4 times more likely to die (HR = 4.25, 95% CI; 1.15-15.7, p = 0.03) compared to patients without comorbidities. Besides, clinically diagnosed patients were almost five times more likely to die (HR = 4.8, 95% CI; 1.43-16, P = 0.01) compared to bacteriologically confirmed TB patients.

Factors significantly associated with reduced survival probability among TB/HIV coinfected patients included, smoking (Yes=0.62, No=0.81: Log Rank p = <0.03), severe malnutrition (severe malnutrition = 0.54, moderate malnutrition = 0.79, normal = 0.84; Log Rank p <0.003), severe illness (severely ill = 0.61, stable = 0.94; Log Rank p <0.001), being previously on TPT (Yes=0.54, No=0.78: Log Rank p = <0.03), having unsuppressed viral load (not suppressed = 0.48, suppressed = 0.66, not done = 0.78, ART Naïve = 0.79; Log Rank = 0.05), previous ART interruption (Yes = 0.56, No = 0.83, ART Naïve = 0.79; Log Rank = 0.04), and comorbidity (no = 0.78, yes = 0.50; Log Rank p <0.01). On multivariable analysis of TB/HIV co-infected patients, smokers were four times more likely to die (HR = 4.05, 95% CI;1.03-16.0, P = 0.04) compared to non-smokers. Also, severely ill patients were almost 6 times more likely to die (HR = 5.84, 95% CI; 1.08-31.6, P = 0.04) compared to clinically stable while severely malnourished patients were five-times more likely to die (HR = 4.56, 95% CI; 1.33-15.6, P = 0.01) compared to normally nourished. Besides, patients with comorbidities were three times more likely to die (HR = 3.04, 95% CI; 1.03-8.97, p = 0.04) compared to patients without comorbidities.

### Causes of death among HIV Negative and HIV Positive TB treatment patients

Mortality audit was conducted for 45 patients who died while on TB treatment. Specific causes of death were categorized based on patients' HIV status and are summarized in Table 4. Majority (72% (TB pneumonia = 44%, lung collapse = 17%, lung fibrosis = 11%)) of HIV negative TB patients died due to TB related lung disease while more than a half (52%) of deaths among HIV positive patients were attributable to advanced HIV disease. Sixteen percent of deaths among HIV positive patients were due to opportunistic infections while 22% of mortality in this group were linked to TB related pneumonia.

**Table 4:** Causes of death among HIV Negative and HIV Positive TB treatment patients

HIV status	Cause of death	Frequency	Percent
HIV Negative	TB Pneumonia	8	44%
	Lung collapse	3	17%
	Lung fibrosis	2	11%
	Status Asthmaticas	1	6%
	Diabetes ketoacidosis	1	6%
	Injury	1	6%
	Liver disease	1	6%
	Lung Cancer	1	6%

HIV Positive	Advanced HIV disease	14	52%
	TB Pneumonia	6	22%
	Liver disease	2	7%
	Cryptococcal Meningitis	1	4%
	Immune reconstitution inflammatory syndrome	1	4%
	Kaposi's Sarcoma	1	4%
	Injury	1	4%
	Pneumocystis jiroveci pneumonia	1	4%

### Conditions contributing to the death among HIV negative and HIV positive TB patients

**Figure 3:**
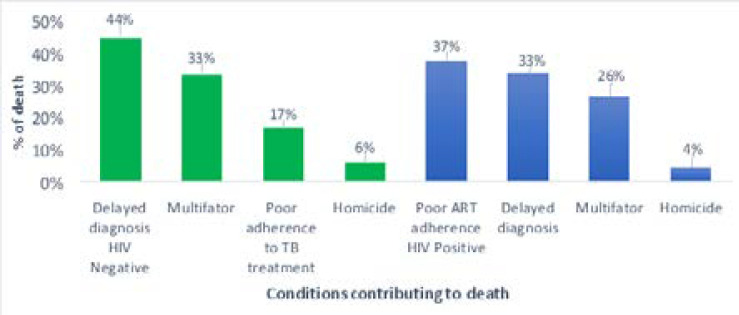
Conditions contributing to mortality

Figure 3 shows conditions contributing to death, but not related to the disease or condition causing it, categorized by HIV status of patients. Forty-four percent of mortality among HIV negative patients were attributable to delayed diagnosis while a third died due multiple patient and health system factors. For the HIV positive mortality was exacerbated by poor adherence to ART (37%) and delayed diagnosis (33%).

## Discussion

Ending TB epidemic requires substantial reduction in TB incidence and mortality 8. This study indicates a high mortality rate among patients on TB treatment predominantly occurring during the intensive phase of treatment. The cumulative incidence of mortality of 15% in the study area is higher than the national average of 6.4%^6^, Kilifi County (5.5%) 31 and Tanzania (3.6%) 15. Similar or higher rates have been observed in other high TB burden countries such as Uganda (15%)^32^, Nigeria (16.6%)^33^ and Zimbabwe (20%)^22^. Although only slightly more than a third of TB patients had one or more comorbid condition( s), patients with comorbidities were associated with significantly reduced survival probability and were independently associated with all-cause mortality including among HIV positive and HIV negative subgroups. Previous studies^16^,^18^,^19^ have similarly demonstrated that comorbidities including diabetes mellitus, malignancies, chronic respiratory conditions, mental health illnesses, liver diseases and chronic kidney diseases are common among TB patients and significantly increase the risk of mortality. Bidirectional negative association between TB and other comorbidities suggests the need for holistic clinical care of TB patients. Universal screening of TB patients for comorbid conditions and vice-versa may result in early detection, better management and improved outcomes. Previous studies^34^,^35^ have indicated that severe illness is not common among TB patients but the associated mortality is high. Whereas the severely ill constituted less than a half (47%) of TB patients in this study, congruent high mortality and reduced survival probability was observed. Severe illness among TB patients, characterized by respiratory rate > 30/min, temperature > 39°C, heart rate > 120/min, inability to walk unaided and severe malnutrition, is likely suggestive of delay in TB diagnosis and treatment. The delay, as observed in this study, may be occasioned by poor patients' health seeking behaviour and/or health care systems gaps. This calls for sustained cost-effective efforts to improve programmatic interventions.

Results of the current study have shown that smoking independently increase the risk of death and significantly reduce survival probability among patients on TB treatment. Studies in Malaysia 20, Brazil 36, India 37 and South Africa 16 as well as systematic reviews 38 have similarly indicated that smoking is a barrier to TB treatment success. Smoking is a risk factors for development of TB disease^37^, reduces lung function and predisposes patients to other conditions such as lung cancer, chronic obstructive airway disease and bronchitis 11,39. Pollack and others 40 demonstrated that smoking is likely to further impair immunity among TB and HIV coinfected patients through its associated 1.5 to 2-fold higher odds of having high HIV viral load. Similarly, smoking was independently associated with increased risk of mortality among TB/HIV coinfected subgroup of patients in this study. Unfortunately, although passive and active smoking were assessed in this study, pack-year index consumed was not quantified. This needs to be explored further. Incorporating smoking control in TB and HIV prevention and control programs may nevertheless contribute to mortality reduction.

Although global reports have shown declining mortality among TB and HIV coinfected patients 1,10, people living with HIV remain a high-risk group for transmission, development of TB disease and dying due to TB^41^. The results of this study have shown higher TB and HIV coinfection rate of 35% compared to national average of 16% 4 and global average of 12% 11. HIV was also independently associated with increased risk of mortality among patients on TB treatment. Previous studies within Kenya 14,17,21 and other high TB burden countries including Tanzania 15, Zimbabwe 22 and South Africa 42 have similarly found HIV to pose a serious mortality risk among TB patients. Predictors of mortality among the HIV positive subgroup included smoking, severe malnutrition, severe illness and comorbidities. Almost all of these factors are suggestive of advanced HIV. Mortality audit also revealed that advanced HIV disease is a major cause of death among TB/HIV coinfected patients while TB pneumonia causes only less than a quarter of deaths. Immune reconstitution inflammatory syndrome and opportunistic infections such as pneumocystis jiroveci pneumonia, cryptococcal meningitis and Kaposi's sarcoma are particularly important causes of mortality among patients on TB treatment while ART interruption and delays in diagnosis appear to play a pivotal role. Previous studies^43^,^44^ have similarly attributed death among TB patients to non-TB causes. Comprehensive HIV care, treatment and prevention presents a unique opportunity for improved TB treatment success. There is also need to further understand reasons for reduced survival probability among TB/HIV coinfected patients previously on TB preventive therapy (TPT). Among the HIV negative group, patients with comorbidities and those clinically diagnosed were associated with increased risk of mortality. From mortality audit, TB related lung disease was the main cause of death. Conditions contributing to death in HIV negative subgroup are consistent with patients' socio-behavioral characteristics and the health system gaps which are modifiable through timely and accurate diagnosis, management of comorbidities, patient education and adherence counselling.
